# Delivery of Mixed-Lineage Kinase Domain-Like Protein by Vapor Nanobubble Photoporation Induces Necroptotic-Like Cell Death in Tumor Cells

**DOI:** 10.3390/ijms20174254

**Published:** 2019-08-30

**Authors:** Lien Van Hoecke, Laurens Raes, Stephan Stremersch, Toon Brans, Juan C. Fraire, Ria Roelandt, Wim Declercq, Peter Vandenabeele, Koen Raemdonck, Kevin Braeckmans, Xavier Saelens

**Affiliations:** 1 VIB-UGent Center for Medical Biotechnology, VIB, 9000 Ghent, Belgium; 2 Department of Biomedical Molecular Biology, Ghent University, 9052 Ghent, Belgium; 3 Laboratory of General Biochemistry & Physical Pharmacy, Ghent University, 9000 Ghent, Belgium; 4 VIB-UGent Center for Inflammation Research, VIB, 9052 Ghent, Belgium; 5 Department of Biochemistry and Microbiology, Ghent University, 9000 Ghent, Belgium

**Keywords:** protein delivery, vapor nanobubble photoporation, necroptotic cell death, mixed-lineage kinase domain-like, apoptotic cell death, caspases

## Abstract

Modern molecular medicine demands techniques to efficiently deliver molecules directly into mammalian cells. As proteins are the final mediators of most cellular pathways, efficient intracellular protein delivery techniques are highly desired. In this respect, photoporation is a promising recent technique for the delivery of proteins directly into living cells. Here, we show the possibility to deliver a model saccharide (FD70) and a model protein (FITC-BSA) into murine B16 melanoma cells by using the vapor nanobubble photoporation technique with an efficiency of 62% and 38%, respectively. Next, we delivered the mixed-lineage kinase domain-like (MLKL) protein, the most terminal mediator of necroptosis currently known, and caspase-8 and -3 protein, which are important proteins in the initiation and execution of apoptosis. A significant drop in cell viability with 62%, 71% and 64% cell survival for MLKL, caspase-8 and caspase-3, respectively, was observed. Remarkably, maximal cell death induction was already observed within 1 h after protein delivery. Transduction of purified recombinant MLKL by photoporation resulted in rapid cell death characterized by cell swelling and cell membrane rupture, both hallmarks of necroptosis. As necroptosis has been identified as a type of cell death with immunogenic properties, this is of interest to anti-cancer immunotherapy. On the other hand, transduction of purified recombinant active caspase-3 or -8 into the tumor cells resulted in rapid cell death preceded by membrane blebbing, which is typical for apoptosis. Our results suggest that the type of cell death of tumor cells can be controlled by direct transduction of effector proteins that are involved in the executioner phase of apoptosis or necroptosis.

## 1. Introduction

Cell death is an evolutionary conserved process in multicellular organisms with far reaching implications in health and disease [1]. Regulated cell death is ultimately controlled by protein activities. Apoptosis, a well-known form of cell death, is the consequence of a caspase signaling cascade involving caspase-8 and -3 [2]. In recent years, it has become increasingly clear that the type of cell death strongly influences the response of other cells in the vicinity of the dying cells. Cell death modalities may range from immunologically silent to a profound immune-stimulating outcome. The latter condition can be highly beneficial in the control of tumors by the patient’s immune system. Indeed, it has been well documented that an immunogenic tumor environment is essential for the clinical success of immunotherapies. To reshape the tumor microenvironment, immune stimulatory molecules [3], blockade of inhibitory cytokines [4] as well as an immune checkpoint blockade [5,6,7] can be introduced at the tumor site to restore immunological fitness. The direct intratumoral injection of mRNA encoding such immune modulatory proteins is an attractive approach. Hewitt and colleagues, for example, delivered an IL-23/IL-36γ/OX40L triplet mRNA mixture combined with checkpoint blockade to successfully reshape the tumor microenvironment [3]. One other way to induce such an immunogenic environment is by eliciting immunogenic cell death that results in the release of damage-associated molecular patterns (DAMPs) and other immune-stimulatory components that can recruit and activate dendritic cells, which are professional antigen-presenting cells. For example, immunogenic apoptosis of neoplastic cells has been reported as a beneficial outcome in response to irradiation, chemotherapies and hypericin-based photodynamic therapy [8,9,10,11,12].

Necroptosis, a regulated form of necrosis, can also have immunogenic properties [13,14]. The necroptotic pathway involves two core proteins, namely receptor interacting protein kinase 3 (RIPK3) and its substrate, mixed-lineage domain-like protein (MLKL) [15,16,17,18,19,20]. Upon its phosphorylation by RIPK3, phosphorylated MLKL oligomerizes and translocates to the plasma membrane, where it induces membrane permeabilization and necroptosis. It is remarkable that many tumor types show genetic and epigenetic changes in the necroptotic pathway. For example, there are cases reported of colon carcinoma, acute myeloid and chronic lymphatic leukemia that show strongly reduced RIPK3 expression levels [21]. Moreover, in pancreatic cancers, reduced MLKL expression is associated with decreased survival of the patient [22,23]. A potential way to restore these aberrations in the necroptotic pathway is by delivery of the executioner protein MLKL, e.g., encoded by in vitro transcribed mRNA, to the tumor cells [24].

An increasing number of mammalian gene products, including intracellular proteins, are now available as purified recombinant proteins [25]. To study the function of such proteins in living cells, the intracellular cytosolic proteins need to be delivered across the plasma membrane into the cytoplasm. There are only a limited number of protein delivery techniques available to date, such as electroporation, microinjection, lipofection or techniques based on cell-penetrating peptides that are fused to the protein of interest [26,27,28,29]. Because of the relatively large size and structural complexity of proteins, the current protein delivery methods often suffer from limited efficacy, which strongly depends on the protein of interest and cell type, or are associated with high cell toxicity. Therefore, efforts are continuously ongoing in search of straightforward broadly applicable approaches for intracellular delivery of macromolecules, including proteins [30,31,32]. One of the newest and most promising transfection technologies is laser-induced vapor nanobubble (VNB) photoporation. It requires cells to be incubated first for a short time with plasmonic gold nanoparticles (AuNPs) that will adsorb onto the cellular plasma membrane. Next, the cells are illuminated with an intense short laser pulse with a wavelength in close proximity to the AuNP plasmon resonance wavelength. Upon light absorption, the AuNPs heat up rapidly, leading to the brisk evaporation of the water surrounding the AuNP surface. The resulting water vapor nanobubble around each AuNP will quickly expand until all thermal energy is consumed, after which, the bubble collapses [33,34]. This causes local shock waves and transient permeabilization of the cell membrane at the location where AuNPs were present. Extracellular molecules in the surrounding cell medium can then diffuse into the cell cytoplasm. The VNB photoporation technology was already shown to allow the efficient delivery of different macromolecules, such as siRNA [35,36,37] and antibodies [38] in a wide variety of cell types, including hard-to-transfect cells such as primary neurons [39] and lymphocytes [36,40].

**Figure 1:**
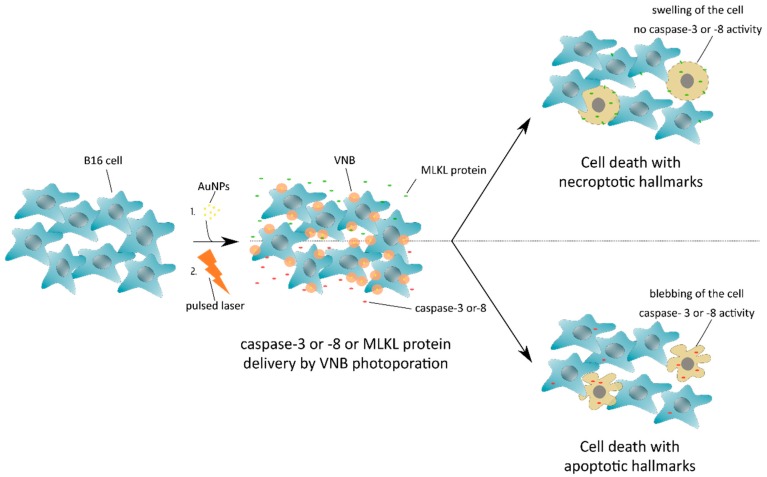
Induction of cell death with necroptotic or apoptotic hallmarks after mixed-lineage kinase domain-like (MLKL) protein or caspase-3/-8 delivery by vapor nanobubble (VNB) photoporation, respectively. B16 cells are first incubated with gold nanoparticles (AuNPs) that adsorb onto the plasma membrane, followed by a nanosecond pulsed laser treatment that results in the formation of vapor nanobubbles (VNBs). Collapse of the expanding VNBs leads to membrane pore formation and allows the extracellular MLKL or caspase -3/-8 protein to diffuse into the cell cytoplasm. After a short period of incubation, the MLKL protein has evoked cell death with necroptotic hallmarks, whereas transduced caspase-3/-8 protein results in apoptosis.

In this study, our aim was to assess if exogenously delivered recombinant proteins associated with two types of cell death, apoptosis and necroptosis, would be sufficient to induce the respective cell death types in B16 melanoma tumor cells. We show here that MLKL protein delivery by VNB photoporation into B16 cells induced cell death with necroptotic hallmarks (swelling of the cells and no caspase activity) (Figure 1). In addition, we demonstrate that VNB photoporation of purified recombinant caspase-3 and -8 into the B16 cells results in rapid blebbing and apoptotic cell death.

## 2. Results

### 2.1. VNB Photoporation Enables Efficient Delivery of 70 kDa FITC-Dextrans into B16 Tumor Cells

**Figure 2:**
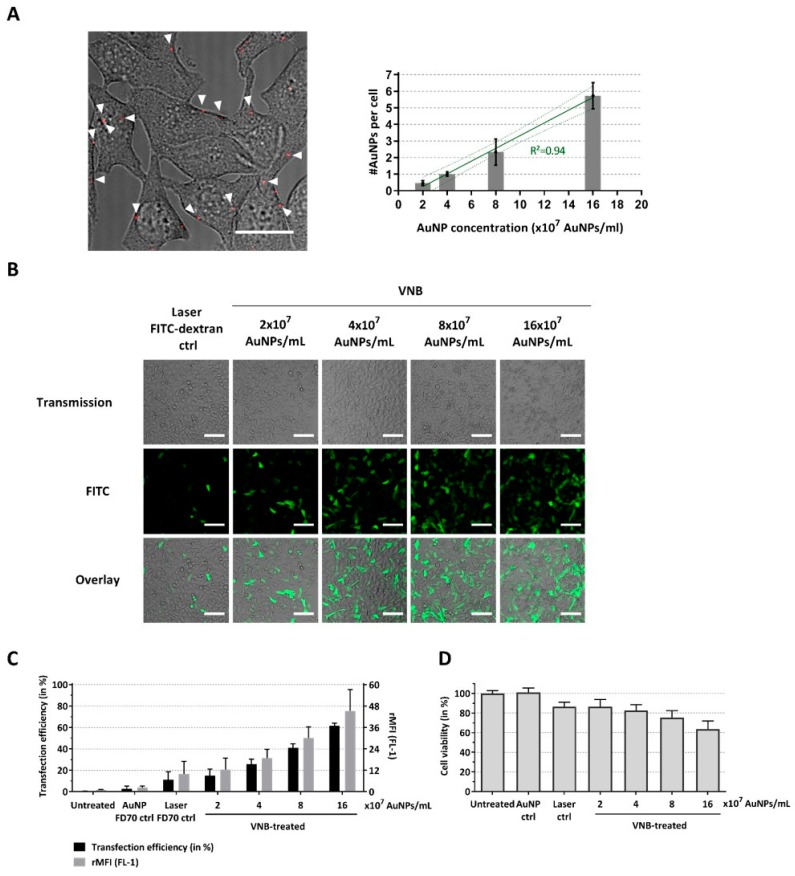
Delivery of FITC-dextran 70 kDa to B16 tumor cells by VNB photoporation. (**A**) B16 cells were incubated with different AuNP concentrations, after which, the excess of AuNPs was washed away. Left: Attachment of AuNPs (concentration: 8 × 10^7^ AuNPs/mL) to the cells visualized by confocal reflection microscopy and overlayed with a light transmission image of the cells (scale bar = 20 µm). Right: The average number of cell-attached AuNPs was quantified for each of the AuNP concentrations using image processing (quantified from >100 cells for each condition). B16 cells were transfected with FITC-dextran 70 kDa (at 2 mg/mL) after incubation with different concentrations of gold nanoparticles (AuNPs). Untreated cells, cells incubated with AuNPs and FITC-dextran, and cells treated only with laser pulses (without AuNPs) were included as controls. (**B**) Confocal images of B16 cells after FITC-dextran delivery (scale bar = 100 µm). (**C**) FITC-dextran transfection efficiency as determined by flow cytometry (*n* = 4, independent experiments). (**D**) Cell viability after photoporation treatment (*n* = 3, independent experiments).

To assess the possibility to use VNB photoporation for efficient macromolecular delivery into mammalian tumor cells (B16 cells), we first applied fluorescently labeled dextrans (FITC-dextran) of 70 kDa (FD70), which are only slightly larger than the proteins of interest (murine MLKL: 56.3 kDa, active murine caspase-3: 32 kDa and active murine caspase-8: 55 kDa). The first step in the photoporation procedure is to incubate cells with AuNPs, which will locally permeabilize the cell membrane upon pulsed laser irradiation. As the concentration of AuNPs that yields optimal transduction results depends on the specific cell type, this parameter was first optimized for the B16 cells. Using confocal reflection microscopy, the number of cell-bound AuNPs after 30 min of incubation was determined for different AuNP concentrations (Figure 2A). As expected, the AuNP concentration correlates positively with the number of cell-adsorbed AuNPs, which ranged from <1 to about 6 AuNPs/cell depending on the concentration used. Next, we performed photoporation experiments for these various AuNP concentrations and confirmed intracellular FD70 delivery by confocal microscopy and, quantitatively, by flow cytometry. From the representative images in Figure 2B, it can be appreciated that the delivery efficiency increases with higher concentrations of AuNPs, which was further confirmed by flow cytometry analysis (Figure 2C). When the highest concentration of AuNPs (16 × 10^7^ AuNPs/mL, ~6 AuNPs/cell) was used, a transduction efficacy of up to 62% positive cells was observed. Higher AuNP concentrations resulted in higher FD70 transduction efficiency, as evident from the gradually increasing mean fluorescence per cell relative to the negative control (rMFI), which is likely related to the formation of more pores in the plasma membrane. However, a higher degree of permeabilization also resulted in increased cell toxicity, as evidenced from the 40% reduction in cell viability when an AuNP concentration of 16 × 10^7^ AuNPs/mL was applied. 

### 2.2. Efficient Protein Delivery in B16 Tumor Cells by VNB Photoporation 

**Figure 3:**
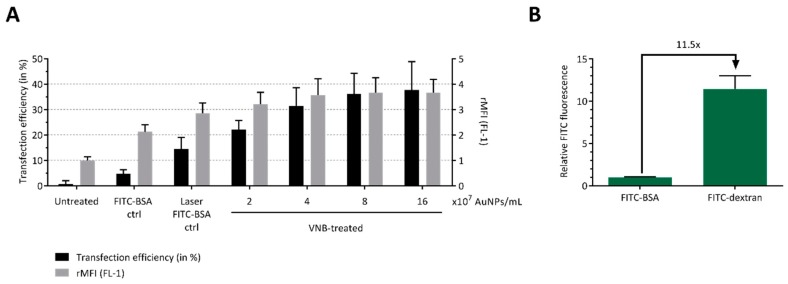
Delivery of FITC-BSA to B16 tumor cells by VNB photoporation. B16 cells were transfected with FITC-BSA (at 2 mg/mL) after incubation with different concentrations of AuNPs. Untreated cells, cells incubated with AuNPs and FITC-BSA, and cells treated only with laser pulses (without AuNPs) were included as controls. (**A**) FITC-BSA transfection efficiency, as determined by flow cytometry (*n* = 3, independent experiments). (**B**) Relative FITC fluorescence of solutions of FITC-BSA (66.5 kDa) and FITC-dextran 70 kDa, measured by fluorimetry at an equal mass concentration of 1 mg/mL (*n* = 3, independent experiments).

In the next step, we assessed whether a model protein could be delivered into B16 cells by photoporation. For this purpose, we selected FITC-conjugated bovine serum albumin (FITC-BSA), which has a molecular weight of 66.5 kDa. Delivery efficiency again increased with increasing AuNP concentrations, reaching up to 38% FITC-BSA positive cells for 16 × 10^7^ AuNP/mL (Figure 3A). On the other hand, the protein transduction appears less efficient compared to FD70 at equal mass concentrations, despite the similar molecular weight. In addition, the relative mean fluorescence intensities (rMFI) of the FITC-BSA transfected cells was lower than that of FD70 transduced cells. This can likely be explained by the relative difference in fluorescence intensity of both compounds. Indeed, measurement of the fluorescent intensity of solutions of FITC-BSA and FITC-dextran 70 kDa at equal mass concentration by fluorimetry shows a >10-fold difference in fluorescent signal (Figure 3B). Based on these results, we can conclude that VNB photoporation enables efficient protein delivery into B16 tumor cells. These data, together with the FD70 transfection results, show that an AuNP concentration of 4 × 10^7^ AuNPs/mL (i.e., approximately 1 AuNP/cell) represents a good balance between optimal transduction efficiency and cell viability and was, therefore, used in all further experiments.

### 2.3. Delivery of Caspase-3/-8 or MLKL by VNB Photoporation Induces Cell Death

We next investigated the functional delivery by photoporation of the necroptotic cell death mediator MLKL and of purified recombinant caspase-3 and caspase-8, well-known executioners and initiators of the apoptotic cell death pathway, respectively. 

**Figure 4:**
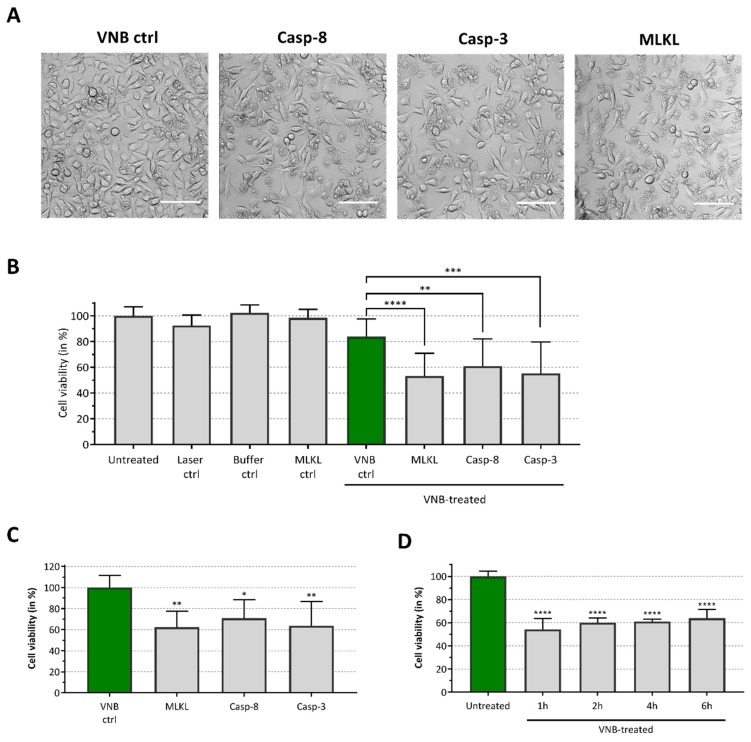
Induction of cell death after caspase-8, caspase-3 and MLKL delivery. B16 cells were transduced with MLKL, caspase-3 or caspase-8 (150 µg/mL) proteins by VNB photoporation (4 × 10^7^ AuNPs/mL). (**A**) Confocal images of B16 cells 6 h after protein delivery (scale bar = 100 µm). (**B**) The graph represents the relative cell viability, as compared to the untreated control (Untreated), 6 h after protein delivery (*n* = 3, independent experiments). B16 cells only illuminated with the pulsed laser (Laser ctrl), cells incubated with the protein storage buffer (Buffer ctrl), cells incubated with the MLKL protein solution (MLKL ctrl) and cells treated by VNB photoporation in absence of proteins (VNB ctrl) were included as controls. (**C**) Relative cell viability, compared to VNB photoporation control (VNB ctrl, green bar), 6 h after protein delivery (*n* = 3, independent experiments). (**D**) Time response analysis of cell death after MLKL delivery. Cell viability was determined 1 h, 2 h, 4 h and 6 h after MLKL delivery, as compared to the untreated control (*n* = 2, independent experiments). * *p* < 0.05; ** *p* < 0.01; *** *p* < 0.001; **** *p* < 0.0001; ns = non-significant (one-way ANOVA with Dunett’s multiple comparison test).

All three proteins were added at a concentration of 150 µg/mL to the photoporation cell medium. After completing the photoporation procedure, the B16 melanoma cells were supplemented with culture medium and placed back in the cell incubator. Six hours after photoporation, a significant decline in viability was detected in the MLKL, caspase-3 and caspase-8 protein groups, as compared to control cells that were photoporated in the absence of any of the three proteins (green bar, Figure 4). This observation was consistent with confocal microscopy images of the cells (Figure 4A) and quantitative CellTiter-Glo^®^ cell viability data (Figure 4B). As cell viability was not affected in the MLKL setting without VNB photoporation (MLKL ctrl, Figure 4A), the detected increased cell death in the MLKL setting was caused by the delivery of the protein via VNB photoporation and not by a possible perturbation of the cell membrane integrity by exogenous MLKL in the cell culture medium. Relative cell viabilities of the protein sample groups, as compared to the photoporation control, show that functional protein delivery resulted in a significant drop in cell viability with 62%, 71% and 64% cell survival for MLKL, caspase-8 and caspase-3, respectively (Figure 4C). These results indicate that VNB photoporation can be used to directly and functionally deliver the protein MLKL, as well as caspases-3 and -8 and that this delivery induces cell death. 

We previously reported that transfection of mRNA encoding MLKL results in B16 melanoma cell death within 16 h after transfection [24]. To probe the time responses of the induction of cell death after photoporation with the MLKL protein itself, we analyzed the kinetics of cell death after MLKL delivery. Cell viability of the B16 cells was determined 1 h, 2 h, 4 h and 6 h after MLKL transfection (Figure 4D). Remarkably, maximal cell death induction was already observed within 1 h after protein delivery and the cell viability remained virtually constant within the following hours. This observation is not surprising considering that VNB photoporation enables direct cytoplasmic delivery of MLKL, which serves as the key player in the final stage for execution of necroptotic cell death [41].

### 2.4. Cell Death Induced by MLKL Delivery Has Necroptotic Hallmarks while Caspase-3/-8 Delivery Induces Apoptotic-Like Cell Death

**Figure 5:**
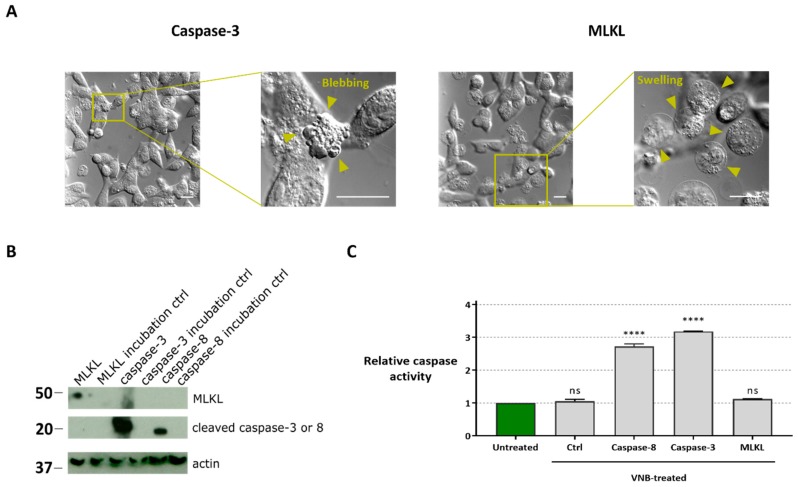
Characterization of cell death modality after protein transfection. (**A**) Differential interference contrast light microscopy visualization of the cell morphology 30 min after caspase-3 and MLKL protein transduction (scale bars = 20 µm). (**B**) Western blot analysis of caspase-3, caspase-8 and MLKL protein in B16 cell lysates with or without (ctrl) VNB photoporation. MLKL, caspase-3, caspase-8 were probed simultaneously using an anti-His detection antibody (exposure 30 min). Actin was visualized using an anti-actin antibody (exposure time 5 min). The cropped blots are presented here, and their full-length blots are included in Supplementary Figure S1. (**C**) Caspase activity in lysates of cells that were transduced with MLKL, caspase-3 or caspase-8 by VNB photoporation. Statistical differences between relative caspase activity in the different samples were determined relative to the untreated control. **** *p* < 0.0001; ns = non-significant (one-way ANOVA with Dunett’s multiple comparison test).

We next evaluated the mode of cell death of B16 cells after MLKL, caspase-3 and caspase-8 protein delivery by photoporation. Transduction of B16 cells with caspase-3 protein resulted in cell death with clear apoptotic features as the formation of apoptotic bodies and membrane blebbing of B16 cells was observed (Figure 5A). In contrast, transduction of the cells with MLKL elicited hallmarks of necroptotic cell death, with clear cell swelling (oncosis) followed by plasma membrane permeabilization (Figure 5A) [42].

Western blot analysis of the MLKL, caspase-3 and caspase-8 in cell lysates prepared 1 h after protein delivery confirmed that all proteins were efficiently delivered to the cells and retained their functional structure after VNB photoporation treatment (Figure 5B). In the incubation controls, in which the protein was added to the cell medium but without photoporation, no protein was detected in the lysates.

To exclude that the apoptotic pathway is involved in the observed cell death after MLKL delivery via photoporation, a caspase activity assay was performed (Figure 5C). In line with the necroptotic cell death phenotype, the caspase activity in B16 cells transduced with the MLKL protein was comparable to the untreated condition. Instead, caspase activity was enhanced compared to untreated cells when caspase-3 or -8 were photoporated into the B16 cells. 

## 3. Discussion

Tumors often comprise a network of both malignant and non-malignant cells. Although various immune effector cells are recruited to the tumor site, in many cases, their anti-tumor functions are downregulated, largely due to tumor-derived immunosuppressive signals. As a consequence, immune cells in the tumor microenvironment fail to exert antitumor effector functions and the tumor escapes from an attack by the host immune system. The immunosuppressive tumor microenvironment is a major obstacle in cancer immunotherapy. Thanks to growing insights into the suppressive mechanism of the tumor microenvironment, possible ways to block tumor escape are currently under investigation, including the delivery at the site of the tumor of immune stimulatory molecules (e.g., OX40 ligand and IL-23 [3]), blockade of inhibitory cytokines (e.g., TGF-β receptor II to capture TGF-β [4]), as well as immune checkpoint blockade (e.g., PD-1 or CTLA-4 [5,6,7]). Recently, it became clear that by eliciting immunogenic cell death (ICD), an immunogenic tumor environment can be created. ICD is a common denominator for different cell death modalities that result in the release of damage-associated molecular patterns (DAMPs). These DAMPs can, together with other immune-stimulatory components, recruit and activate DCs in the tumor microenvironment [43,44,45]. It is shown that tumor cells succumbing from irradiation, chemotherapeutics and hypericin-based photodynamic therapy undergo immunogenic apoptosis [8,9,10,11,12]. In addition, necroptosis is considered as a type of cell death with immunogenic properties [13,14].

In this study, we show that caspase-3 or -8, or MLKL protein delivery by VNB photoporation induces, respectively, apoptotic and necroptotic cell death in murine B16 melanoma tumor cells. The B16 tumor cell line is frequently used as a model for human melanoma. VNB photoporation represents a very promising physical transfection technology, already shown to enable gentle intracellular delivery of different types of macromolecules (e.g., siRNA) in a wide variety cell types, such as cancer cell lines [35,46], primary lymphocytes [36,40] and neuronal cells [39]. By combination of absorbing gold nanoparticles and a weakly focused laser beam, the technology also allows simultaneous intracellular delivery of macromolecules in a large number of cells, while maintaining a high level of spatial control [35,47]. First, we showed that VNB photoporation enables efficient delivery of FITC-dextrans and FITC-BSA into B16 tumor cells. For this, B16 cells were incubated with different concentrations of gold nanoparticles (AuNPs), followed by VNB generation by pulsed laser irradiation in the presence of FITC-dextrans or FITC-BSA molecules. Considering that only a few gold nanoparticles adsorb to each cell and the arising VNBs only affect neighboring structures very locally, damage to nearby molecules is possible but negligible on the total molecule ensemble level. In general, we found that the higher the used AuNP concentration, the higher the transduction efficacy and, accordingly, the lower the cell viability. Delivery of caspase-3 by gold-nanoparticle-mediated laser transfection (GNOME) has been reported in ZMTH3 cells (originating from a pleomorphic mammary adenoma), by Heinemann et al. [37]. The authors were able to show that the transduction of ZMTH3 cells with caspase-3 resulted in cell death. Here, we confirmed that transduction of the caspase-3 protein in B16 tumor cells results in the induction of cell death. In addition, we have shown that the evoked cell death has apoptotic features, i.e., cell blebbing and intracellular caspase activity. Furthermore, by transducing B16 tumor cells with caspase-8 protein, another member of the cysteine protease family which is implicated in apoptosis, cell death with apoptotic features was induced.

Next to apoptosis, there are other kinds of regulated cell death modalities, each with their own specific features. For example, necroptosis is an interesting type of cell death as it has immunogenic properties [13,14,24]. Necroptotic cells release damage-associated molecular patterns (DAMPs) and other immune-stimulatory components that, in an organism, can recruit immune cells to the dying cell. This has very interesting implications in the context of the induction of an immune response against tumor cells. Nonetheless, induction of necroptosis in tumor cells represents a major challenge, as many tumor types display genetic and epigenetic alterations in the pathway, leading to necroptosis [48]. For this reason, we opted to deliver the MLKL protein as a downstream executioner of the necroptotic pathway. By performing protein transduction, manipulation of cells at the genetic level is circumvented. We showed that it is possible to induce cell death with necroptotic features, such as cell swelling and absence of caspase activity, via MLKL protein delivery by means of VNB photoporation in B16 cells. As MLKL protein is the final mediator of necroptosis, direct intracellular delivery of the protein by VNB photoporation very rapidly resulted in cell death. In conclusion, we demonstrated that direct transduction of proteins involved in the executioner phase of either apoptosis or necroptosis allows accurate and rapid control of the cell death modality.

## 4. Materials and Methods

### 4.1. Materials

Cationic gold nanoparticles (AuNPs) with a core size of 60 nm were synthetized in-house using the Turkevich method, as reported before [40,49]. The AuNPs had a zeta potential of at least + 30 mV, as verified by dynamic light scattering (Malvern Instruments, Worcestershire, UK). FITC-dextran 70 kDa and FITC-BSA were purchased from Sigma-Aldrich (Bornem, Belgium), dissolved in DPBS- (without MgCl_2_ and CaCl_2_) at concentrations of, respectively, 50 mg/mL and 25 mg/mL and stored at 4 °C until further use. The recombinant mouse MLKL protein (recombinant 6His, N-terminus) (full length) was purchased from LifeSpan BioSciences (Seattle, Washington, USA) (catalog number/lot number: LS-G23454/132989) and dissolved in a Tris, 50% glycerol buffer. The recombinant mouse caspase-3 (Casp-3, recombinant 6His, N-terminus) (full length) and caspase-8 (Casp-8, recombinant 6His, N-terminus) (full length) proteins were made in-house.

### 4.2. Cell Lines and Culture Conditions

Cells were cultured in Dulbecco’s modified Eagle’s medium supplemented with 10% of fetal calf serum, 2 mM of L-glutamine, 0.4 mM of Na-pyruvate, non-essential amino acids, 100 U:mL of penicillin and 0.1 mg/mL of streptomycin at 37 °C in a humidified atmosphere containing 5% CO_2_. Murine tumor cells used were melanoma cell lines (B16). These cells were purchased from the American Type Culture collection (ATCC, LGC Standards Sarl, Molsheim Cedex, France) and cultured in conditions specified by the manufacturer.

### 4.3. Measurement of FITC Fluorescence

FITC-dextrans and FITC-BSA were diluted in DPBS- to a final concentration of 1 mg/mL, and 100µL of these dilutions was transferred to a black 96-well plate (Greiner Bio-One). FITC fluorescence was measured using a Victor3 multilabel reader (Perkin Elmer, Boston, MA, USA) with an excitation/emission wavelength of 485/535 nm and measurement time of 0.1 sec.

### 4.4. Visualization and Quantification of AuNP Attachment

B16 cells (30 × 10^3^ cells/well) were seeded in a glass-bottom 96-well plate (Greiner Bio-One, Frickenhausen, Germany) and allowed to attach overnight at 37 °C in a humidified atmosphere containing 5% CO_2_. The cells were incubated for 30 min with different concentrations of AuNPs (37 °C and 5% CO_2_), followed by a wash step with DPBS- to remove unbound AuNPs. Visualization of cell-attached AuNPs was performed by confocal reflection microscopy (C2, Nikon Benelux, Brussels, Belgium) with a 60× water immersion lens at room temperature. Image processing was carried out using the ImageJ software package (FIJI), and involved masking the AuNP scattering and dilation of the obtained mask. By counting the number of AuNPs and cells in each image, the average number of AuNPs per cell was quantified.

### 4.5. Transfection by Vapor Nanobubble (VNB) Photoporation

For FITC-dextran or protein delivery by VNB photoporation, B16 cells (30 × 10^3^ cells/well) were seeded in a 96-well plate and allowed to attach overnight at 37 °C in a humidified atmosphere containing 5% CO_2_. First, the cells were incubated for 30 min with AuNPs at a concentration dependent on the specific experiment, followed by a washing step with DPBS- to remove unbound AuNPs. Prior to the pulsed laser treatment, the specific cargo was diluted in culture medium (FITC-dextran, FITC-BSA) or Opti-MEM™ (MLKL, Casp-8, Casp-3) to the appropriate concentration and added to the cells. For generation of VNBs, a homemade set-up was used [35,49]. This set-up consists of a pulsed laser (~7ns) tuned at a wavelength of 561 nm (Opolette HE 355 LD, OPOTEK Inc) that was applied for illumination of the cell-bound AuNPs and subsequent generation of VNBs. A laser fluence of 1.9 J/cm², i.e., above the VNB generation threshold [35,36], was used to assure the efficient formation of VNBs. After treatment at room temperature, the cells were washed twice with DPBS- and supplied with fresh culture medium (FITC-dextran, FITC-BSA), or immediately supplemented with fresh culture medium (MLKL, Casp-8, Casp-3).

### 4.6. Viability Assay

B16 cells were treated using different concentrations of AuNPs, as described previously. After 2 h of incubation, cell viability was determined using a CellTiter-Glo^®^ luminescence cell viability assay (Promega, Leiden, The Netherlands). For this, an equal volume of CellTiter-Glo^®^ reagent was added to the culture medium on the cells (at room temperature). The plate was shaken for 10 min using an orbital shaker (100 rpm) and the content of each well was transferred to an opaque 96-well plate. The luminescent signal was allowed to stabilize for 10 min, after which, the luminescence in each well was recorded by a GloMax™ Luminometer (Promega, Leiden, The Netherlands).

### 4.7. Confocal Microcopy

Visualization of B16 cells transfected with FITC-dextran 70 kDa, caspase-8, caspase-3 or MLKL was performed using confocal laser scanning microscopy (C2, Nikon Benelux, Brussels, Belgium) and a 10× objective lens. Uptake of FITC dextran 70 kDa was detected using a 488 nm laser as excitation source.

### 4.8. Flow Cytometry

To evaluate the transfection efficiency after photoporation by flow cytometry, the B16 cells were detached by trypsin/EDTA (0.25%), centrifuged (500× *g*, 5 min) and resuspended in flow buffer (DPBS-, 0.1% Sodium Azide, 1% Bovine Serum Albumine). Flow cytometry was performed using a CytoFLEX (Beckman Coulter, Suarlée, Belgium) flow cytometer and FlowJo™ software (Treestar Inc.) was used for data analysis.

### 4.9. DIC Visualization of Cell Death

For enhanced contrast visualization of cell death, B16 cells (100 × 10^3^ cells/dish) were seeded in 50 mm γ-irradiated glass-bottom dishes (MatTek) and allowed to attach overnight. The cells were transfected with MLKL, caspase-8 or caspase-3, as described above. After 30 min of incubation, cell death was visualized by differential interference contrast imaging using a 60× oil immersion objective lens (NA = 1.4, Nikon). Background correction was applied to the resulting images.

### 4.10. Western Blot

B16 cells (30 × 10^3^ cells/well) were seeded in a 96-well plate and transfected with MLKL, caspase-8 and caspase-3 24 h later, as described above. After photoporation of B16 cells, cells were washed to remove recombinant proteins that were not taken up by the cells via photoporation. One hour after photoporation, cells were lysed and lysates were separated using sodium dodecyl sulfate-polyacrylamide gel electrophoresis (10% acrylamide). MLKL, caspase-8 and caspase-3 were visualized by Western blotting with anti-His antibody (1000× dilution) (Bio-rad AbD Serotec, Kidlington, United Kingdom catalog number: MCA1396).

### 4.11. Caspase Activity

To analyze caspase activity, a FLUOstar OMEGA (BMG Labtech, Ortenberg, Germany) assay was performed. Therefore, B16 cells (30 × 10^3^ cells/well) were seeded in a 96-well plate and photoporated 24 h later. After photoporation, cells were washed and lysed in CFS-buffer (pH 7.5) with 10 mM DTT to sustain caspase activity. CFS-buffer contained 10 mM Hepes, 220 mM mannitol, 68 mM sucrose, 2 mM NaCl, 2 mM MgCl_2_, and 2.5 mM KH_2_PO_4_. Two hours after photoporation, 33 mM of Ac-DEVD-AMC and Ac-IETD-AMC were added to the cells. Caspase activity was measured by cleavage of Av-DEVD-AMC and Ac-IETD-AMC into fluorescent 7-amino-4methylcoumarin (AMC) (excitation 355 nm, emission 460 nm).

### 4.12. Statistical Analysis

All statistical analyses are performed using the GraphPad Prism 8 software (La Jolla). Data sets are represented as mean ± standard deviation.

## 5. Conclusions

In conclusion, we show that by using VNB photoporation it is feasible to deliver, in vitro, caspase-3/-8 or MLKL protein to murine B16 tumor cells. Such an intervention induces cell death with respectively apoptotic or necroptotic characteristics.

## Supplementary Materials

Supplementary materials can be found at https://www.mdpi.com/1422-0067/20/17/4254/s1. Figure S1. Full-length blots corresponding to Figure 5B.

## Abbreviations

MLKL	Mixed-lineage kinase domain-like
RIPK	receptor interacting protein kinase
VNB	vapor nanobubble
AuNPs	gold nanoparticles
FD70	FITC-dextran of 70 kDa
GNOME	gold-nanoparticle-mediated laser transfection

## References

[1] Labi V., Erlacher M. (2015). How cell death shapes cancer. Cell Death Dis..

[2] Tang D., Kang R., Berghe T.V., Vandenabeele P., Kroemer G. (2019). The molecular machinery of regulated cell death. Cell Res..

[3] Hewitt S.L., Bai A., Bailey D., Ichikawa K., Zielinski J., Karp R., Apte A., Arnold K., Zacharek S.J., Iliou M.S. (2019). Durable anticancer immunity from intratumoral administration of IL-23, IL-36gamma, and OX40L mRNAs. Sci. Transl. Med..

[4] Van der Jeught K., Joe P.T., Bialkowski L., Heirman C., Daszkiewicz L., Liechtenstein T., Escors D., Thielemans K., Breckpot K. (2014). Intratumoral administration of mRNA encoding a fusokine consisting of IFN-beta and the ectodomain of the TGF-beta receptor II potentiates antitumor immunity. Oncotarget.

[5] Hugo W., Zaretsky J.M., Sun L., Song C., Moreno B.H., Hu-Lieskovan S., Berent-Maoz B., Pang J., Chmielowski B., Cherry G. (2016). Genomic and Transcriptomic Features of Response to Anti-PD-1 Therapy in Metastatic Melanoma. Cell.

[6] Ribas A., Dummer R., Puzanov I., VanderWalde A., Andtbacka R.H.I., Michielin O., Olszanski A.J., Malvehy J., Cebon J., Fernandez E. (2017). Oncolytic Virotherapy Promotes Intratumoral T Cell Infiltration and Improves Anti-PD-1 Immunotherapy. Cell.

[7] Tumeh P.C., Harview C.L., Yearley J.H., Shintaku I.P., Taylor E.J., Robert L., Chmielowski B., Spasic M., Henry G., Ciobanu V. (2014). PD-1 blockade induces responses by inhibiting adaptive immune resistance. Nature.

[8] Obeid M., Tesniere A., Ghiringhelli F., Fimia G.M., Apetoh L., Perfettini J.L., Castedo M., Mignot G., Panaretakis T., Casares N. (2007). Calreticulin exposure dictates the immunogenicity of cancer cell death. Nat. Med..

[9] Garg A.D., Krysko D.V., Verfaillie T., Kaczmarek A., Ferreira G.B., Marysael T., Rubio N., Firczuk M., Mathieu C., Roebroek A.J. (2012). A novel pathway combining calreticulin exposure and ATP secretion in immunogenic cancer cell death. EMBO J..

[10] Galluzzi L., Kepp O., Kroemer G. (2012). Enlightening the impact of immunogenic cell death in photodynamic cancer therapy. EMBO J..

[11] Galluzzi L., Kepp O., Kroemer G. (2013). Immunogenic cell death in radiation therapy. Oncoimmunology.

[12] Kroemer G., Galluzzi L., Kepp O., Zitvogel L. (2013). Immunogenic cell death in cancer therapy. Annu. Rev. Immunol..

[13] Yatim N., Jusforgues-Saklani H., Orozco S., Schulz O., Barreira da Silva R., Reis e Sousa C., Green D.R., Oberst A., Albert M.L. (2015). RIPK1 and NF-kappaB signaling in dying cells determines cross-priming of CD8(+) T cells. Science.

[14] Aaes T.L., Kaczmarek A., Delvaeye T., De Craene B., De Koker S., Heyndrickx L., Delrue I., Taminau J., Wiernicki B., De Groote P. (2016). Vaccination with Necroptotic Cancer Cells Induces Efficient Anti-tumor Immunity. Cell Rep..

[15] Sun L., Wang H., Wang Z., He S., Chen S., Liao D., Wang L., Yan J., Liu W., Lei X. (2012). Mixed lineage kinase domain-like protein mediates necrosis signaling downstream of RIP3 kinase. Cell.

[16] Zhao J., Jitkaew S., Cai Z., Choksi S., Li Q., Luo J., Liu Z.G. (2012). Mixed lineage kinase domain-like is a key receptor interacting protein 3 downstream component of TNF-induced necrosis. Proc. Natl. Acad. Sci. USA.

[17] Murphy J.M., Czabotar P.E., Hildebrand J.M., Lucet I.S., Zhang J.G., Alvarez-Diaz S., Lewis R., Lalaoui N., Metcalf D., Webb A.I. (2013). The pseudokinase MLKL mediates necroptosis via a molecular switch mechanism. Immunity.

[18] Wang H., Sun L., Su L., Rizo J., Liu L., Wang L.F., Wang F.S., Wang X. (2014). Mixed lineage kinase domain-like protein MLKL causes necrotic membrane disruption upon phosphorylation by RIP3. Mol. Cell.

[19] Dondelinger Y., Declercq W., Montessuit S., Roelandt R., Goncalves A., Bruggeman I., Hulpiau P., Weber K., Sehon C.A., Marquis R.W. (2014). MLKL compromises plasma membrane integrity by binding to phosphatidylinositol phosphates. Cell Rep..

[20] Cai Z., Jitkaew S., Zhao J., Chiang H.C., Choksi S., Liu J., Ward Y., Wu L.G., Liu Z.G. (2014). Plasma membrane translocation of trimerized MLKL protein is required for TNF-induced necroptosis. Nat. Cell Biol..

[21] Moriwaki K., Bertin J., Gough P.J., Orlowski G.M., Chan F.K. (2015). Differential roles of RIPK1 and RIPK3 in TNF-induced necroptosis and chemotherapeutic agent-induced cell death. Cell Death Dis..

[22] Colbert L.E., Fisher S.B., Hardy C.W., Hall W.A., Saka B., Shelton J.W., Petrova A.V., Warren M.D., Pantazides B.G., Gandhi K. (2013). Pronecrotic mixed lineage kinase domain-like protein expression is a prognostic biomarker in patients with early-stage resected pancreatic adenocarcinoma. Cancer.

[23] He L., Peng K., Liu Y., Xiong J., Zhu F.F. (2013). Low expression of mixed lineage kinase domain-like protein is associated with poor prognosis in ovarian cancer patients. OncoTargets Ther..

[24] Van Hoecke L., Van Lint S., Roose K., Van Parys A., Vandenabeele P., Grooten J., Tavernier J., De Koker S., Saelens X. (2018). Treatment with mRNA coding for the necroptosis mediator MLKL induces antitumor immunity directed against neo-epitopes. Nat. Commun..

[25] McKenzie E.A., Abbott W.M. (2018). Expression of recombinant proteins in insect and mammalian cells. Methods.

[26] Ford K.G., Souberbielle B.E., Darling D., Farzaneh F. (2001). Protein transduction: An alternative to genetic intervention?. Gene Ther..

[27] Morris M.C., Depollier J., Mery J., Heitz F., Divita G. (2001). A peptide carrier for the delivery of biologically active proteins into mammalian cells. Nat. Biotechnol..

[28] Todorova R. (2009). Estimation of methods of protein delivery into mammalian cells—A comparative study by electroporation and bioporter assay. Prikl. Biokhim. Mikrobiol..

[29] Zassler B., Blasig I.E., Humpel C. (2005). Protein delivery of caspase-3 induces cell death in malignant C6 glioma, primary astrocytes and immortalized and primary brain capillary endothelial cells. J. Neurooncol..

[30] Stewart M.P., Langer R., Jensen K.F. (2018). Intracellular Delivery by Membrane Disruption: Mechanisms, Strategies, and Concepts. Chem. Rev..

[31] Zhang Y., Roise J.J., Lee K., Li J., Murthy N. (2018). Recent developments in intracellular protein delivery. Curr. Opin. Biotechnol..

[32] Bruce V.J., McNaughton B.R. (2017). Inside Job: Methods for Delivering Proteins to the Interior of Mammalian Cells. Cell Chem. Biol..

[33] Xiong W., Liu Y., Jiang L.J., Zhou Y.S., Li D.W., Jiang L., Silvain J.F., Lu Y.F. (2016). Laser-Directed Assembly of Aligned Carbon Nanotubes in Three Dimensions for Multifunctional Device Fabrication. Adv. Mater..

[34] Lapotko D. (2009). Optical excitation and detection of vapor bubbles around plasmonic nanoparticles. Opt. Express.

[35] Xiong R., Raemdonck K., Peynshaert K., Lentacker I., De Cock I., Demeester J., De Smedt S.C., Skirtach A.G., Braeckmans K. (2014). Comparison of gold nanoparticle mediated photoporation: Vapor nanobubbles outperform direct heating for delivering macromolecules in live cells. ACS Nano.

[36] Wayteck L., Xiong R., Braeckmans K., De Smedt S.C., Raemdonck K. (2017). Comparing photoporation and nucleofection for delivery of small interfering RNA to cytotoxic T cells. J. Control. Release.

[37] Heinemann D., Kalies S., Schomaker M., Ertmer W., Murua Escobar H., Meyer H., Ripken T. (2014). Delivery of proteins to mammalian cells via gold nanoparticle mediated laser transfection. Nanotechnology.

[38] Liu J., Xiong R., Brans T., Lippens S., Parthoens E., Zanacchi F.C., Magrassi R., Singh S.K., Kurungot S., Szunerits S. (2018). Repeated photoporation with graphene quantum dots enables homogeneous labeling of live cells with extrinsic markers for fluorescence microscopy. Light Sci. Appl..

[39] Xiong R., Verstraelen P., Demeester J., Skirtach A.G., Timmermans J.P., De Smedt S.C., De Vos W.H., Braeckmans K. (2018). Selective Labeling of Individual Neurons in Dense Cultured Networks with Nanoparticle-Enhanced Photoporation. Front. Cell. Neurosci..

[40] Raes L., Hecke C.V., Michiels J., Stremersch S., Fraire J.C., Brans T., Xiong R., De Smedt S., Vandekerckhove L., Raemdonck K. (2019). Gold Nanoparticle-Mediated Photoporation Enables Delivery of Macromolecules over a Wide Range of Molecular Weights in Human CD4+ T Cells. Crystals.

[41] Chen D., Yu J., Zhang L. (2016). Necroptosis: An alternative cell death program defending against cancer. Biochim. Biophys. Acta.

[42] Majno G., Joris I. (1995). Apoptosis, oncosis, and necrosis. An overview of cell death. Am. J. Pathol..

[43] Hackl H., Charoentong P., Finotello F., Trajanoski Z. (2016). Computational genomics tools for dissecting tumour-immune cell interactions. Nat. Rev. Genet..

[44] Krysko O., Love Aaes T., Bachert C., Vandenabeele P., Krysko D.V. (2013). Many faces of DAMPs in cancer therapy. Cell Death Dis..

[45] Krysko D.V., Garg A.D., Kaczmarek A., Krysko O., Agostinis P., Vandenabeele P. (2012). Immunogenic cell death and DAMPs in cancer therapy. Nat. Rev. Cancer.

[46] Xiong R., Joris F., Liang S., De Rycke R., Lippens S., Demeester J., Skirtach A., Raemdonck K., Himmelreich U., De Smedt S.C. (2016). Cytosolic Delivery of Nanolabels Prevents Their Asymmetric Inheritance and Enables Extended Quantitative in Vivo Cell Imaging. Nano Lett..

[47] Xiong R., Drullion C., Verstraelen P., Demeester J., Skirtach A.G., Abbadie C., De Vos W.H., De Smedt S.C., Braeckmans K. (2017). Fast spatial-selective delivery into live cells. J. Control. Release.

[48] Krysko O., Aaes T.L., Kagan V.E., D’Herde K., Bachert C., Leybaert L., Vandenabeele P., Krysko D.V. (2017). Necroptotic cell death in anti-cancer therapy. Immunol. Rev..

[49] Vermeulen L.M.P., Fraire J.C., Raes L., De Meester E., De Keulenaer S., Van Nieuwerburgh F., De Smedt S., Remaut K., Braeckmans K. (2018). Photothermally Triggered Endosomal Escape and Its Influence on Transfection Efficiency of Gold-Functionalized JetPEI/pDNA Nanoparticles. Int. J. Mol. Sci..

