# Supplementary material for: Delivery of Mixed-Lineage Kinase Domain-Like Protein by Vapor Nanobubble Photoporation Induces Necroptotic-Like Cell Death in Tumor Cells
Source: Int J Mol Sci. 2019 Aug 30;20(17):4254. doi: 10.3390/ijms20174254 (PMC6747363; doi:10.3390/ijms20174254)
Supplement: Supplementary file 1 [file ijms-20-04254-s001.pdf]

Article

Delivery of Mixed-Lineage Kinase Domain-Like Protein by Vapor Nanobubble Photoporation Induces Necroptotic-Like Cell Death in Tumor Cells

Lien Van Hoecke [1,2,†], Laurens Raes [3,†], Stephan Stremersch [3], Toon Brans [3], Juan C. Fraire [3], Ria Roelandt [2,4], Wim Declercq [2,4], Peter Vandenabeele [2,5], Koen Raemdonck [3], Kevin Braeckmans [3] and Xavier Saelens [1,5,*]

1 VIB-UGent Center for Medical Biotechnology, VIB, 9000 Ghent, Belgium
2 Department of Biomedical Molecular Biology, Ghent University, 9052 Ghent, Belgium
3 Laboratory of General Biochemistry & Physical Pharmacy, Ghent University, 9000 Ghent, Belgium.
4 VIB-UGent Center for Inflammation Research, VIB, 9052 Ghent, Belgium
5 Department of Biochemistry and Microbiology, Ghent University, 9000 Ghent, Belgium
* Correspondence: Xavier.saelens@vib-ugent.be
† These authors contributed equally to this work.

A

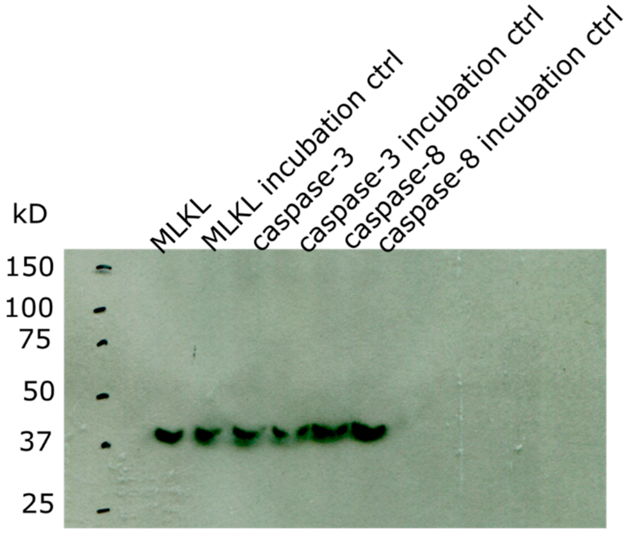

B

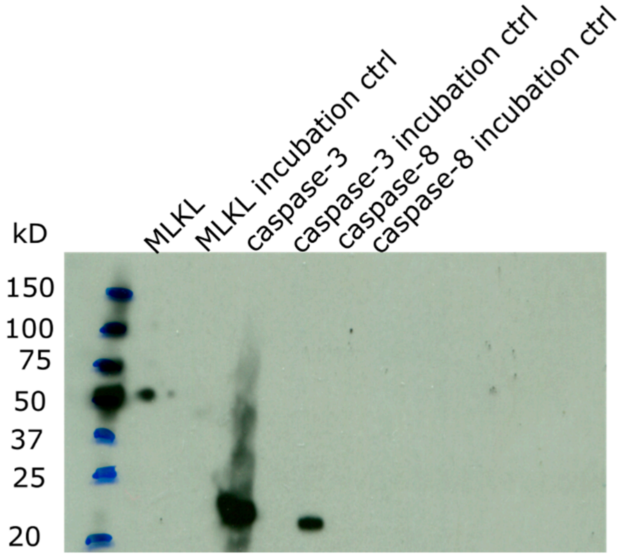

Figure S1. Full-length blots corresponding to Figure 5B. (**A**) Full-length blot of the detection of actin using anti-actin as the primary antibody. The exposure time was 5 minutes. (**B**) Full-length blot of the simultaneous detection of MLKL, caspase-3 and caspase-8 using anti-His antibody as the primary antibody. The exposure time was 30 minutes. The kD values to the left of the blots represent the molecular mass of the bands of the individual proteins from the precision Plus Protein All Blue Standards of Biorad ®.
